# Human Herpes Virus 6-Associated Meningitis in an Immunocompetent Adult With a History of Plaque Psoriasis: A Case Report

**DOI:** 10.7759/cureus.50241

**Published:** 2023-12-09

**Authors:** Gennifer Makhoul Wahbah, Nnedindu Asogwa, Joanne C Ling, Alaukika Agarwal, Elizabeth Rimsky, Allison Glaser

**Affiliations:** 1 Internal Medicine, Staten Island University Hospital, New York, USA

**Keywords:** autoimmune, neurotropic, psoriasis, meningitis, human herpes virus

## Abstract

Human herpes virus-6 (HHV-6) is a common cause of viral infection in humans, recognized for causing exanthema subitem during the first two years of life. Chronic inflammatory states in different autoimmune diseases can be associated with an immunosuppressed microenvironment. We report a case of HHV-6-associated meningitis in a patient with psoriasis. A 36-year-old man with a history of psoriasis presented with worsening bifrontal headache as well as painful oral lesions on the tongue and soft palate. Computed tomographic (CT) scan of the brain was nondiagnostic. Cerebrospinal fluid (CSF) analysis was diagnostic for HHV-6 infection and was treated with intravenous ganciclovir for two weeks. This case report highlights the emergence of HHV-6 infections in people with underlying mild immune disorders, such as psoriasis. Plaque psoriasis, such as in this patient, could be related to viral infections not typically seen among adults.

## Introduction

Human herpes virus-6 (HHV-6) is a double-helix DNA virus, classified among nine known herpesviruses that primarily infect humans [1]. HHV-6 is particularly notable for causing exanthema subitum (also known as roseola infantum or sixth disease). Exanthema subitum is a benign febrile illness acquired during the first two years of life [2]. According to available data, the seroprevalence of HHV-6 among adults in developed countries exceeds 70% [3]. The virus was first isolated in 1986 from the blood of patients with acquired immunodeficiency syndrome (AIDS) and lymphoproliferative diseases [4]. HHV-6 infections in adults are rare and can be predominantly found in immunocompromised individuals, especially patients with a history of human immunodeficiency virus (HIV) infection [5]. Current literature suggests that HHV-6 is neurotropic, exhibiting a predilection for the nervous system, causing a variety of disorders including multiple sclerosis and encephalitis/meningitis in immunocompetent adults [6].

Psoriasis, an autoimmune disorder, is defined by the presence of distinguishing plaques and desquamating skin lesions across the body surface [7]. Literature has shown that even mild cases of psoriasis can be associated with elevated levels of inflammatory markers, such as C-reactive protein, which invariably is a marker of ongoing non-infectious inflammatory processes [8]. Chronic inflammatory states can be associated with an abnormal immunosuppressed microenvironment. This allows for increased tumor formation and more opportunistic infections with less common organisms [9]. Of the few cases of HHV-6 meningitis/encephalitis in adults, none have been reported in the setting of an ongoing immune disorder such as psoriasis. In this case report, we explore a case of HHV-6 meningitis in an adult male patient with a history of untreated skin psoriasis after a positive sick exposure.

## Case presentation

A 36-year-old male presented to the emergency department (ED) with a chief complaint of worsening bifrontal headache. Two weeks prior to presentation, the patient had episodes of chills, fevers, and sore throat. During this time, he self-treated with over-the-counter acetaminophen 325mg. As his symptoms progressed, painful oral lesions developed on the tongue and soft palate. Seeking medical attention, he visited an urgent care center where he was prescribed a course of oral valacyclovir, of which he only took two doses.

The onset of a new bifrontal throbbing headache, accompanied by nausea and intermittent photophobia, led him to seek further care in the ED. The patient described recent sick exposure to his one-year-old son whose diagnosis was presumed to be Coxsackie virus infection. His past medical history was significant for plaque psoriasis. Vital signs in the ED showed a temperature of 36.4 °C, blood pressure of 133/80 mmHg, heart rate of 92 beats per minute, respiratory rate of 18 breaths per minute, and oxygen saturation of 99% on room air. Physical examination revealed multiple aphthous ulcers in the mouth, limited to the tongue and soft palate. The neck exam was supple and Brudzinski’s sign was negative. Skin examination showed no active psoriatic lesions. Initial laboratory workup and computed tomography (CT) of the head without IV contrast were unremarkable. The patient was subsequently discharged from the ED.

Two days later, the patient presented a second time to the ED for persistent symptoms and unremitting bifrontal headache. Vital signs on this ED visit showed a temperature of 36.9 °C, blood pressure of 141/72 mmHg, heart rate of 90 beats per minute, respiratory rate of 18 breaths per minute, and oxygen saturation of 98% on room air. Physical examination was significant for the persistence of oral aphthous ulcers on the tongue and soft palate. No fundus examination was performed. Laboratory testing revealed a white blood cell 13.35 k/uL count with 62.8% neutrophils and 30.9% lymphocytes. Repeat CT of the head without IV contrast was not significant for any new findings. A lumbar puncture performed showed 468 total nucleated cell count/uL (54% neutrophil percentage, 40% lymphocyte percentage, 6% monocytes percentage), 156 red blood cell count/uL, 68mg/dl proteins and 55mg/dl glucose in the cerebrospinal fluid (CSF). The patient was started on empirical treatment for meningitis, and received in the ED one dose of 1350 mg acyclovir IV, one dose of 2000 mg vancomycin IV daily, and one dose of 10mg dexamethasone IV. CSF polymerase chain reaction (PCR) was diagnostic for HHV-6 infection. The patient was admitted to the hospital with a primary diagnosis of HHV-6 meningitis. Parenteral antibiotics and steroids were stopped and subsequently started on ganciclovir 5mg/kg IV every 12 hours with monitoring of symptoms. The hospital course was uncomplicated, and the patient was discharged home after a three-day hospital stay and was instructed to take a 21-day course of oral valganciclovir every 12 hours. Four days after being discharged home, the patient noted a maculopapular erythematous rash of >1cm in size located at the medial aspect midcalf level of the left lower extremity that self-resolved over one week with no interventions. 

## Discussion

HHV-6 is a neurotropic and lymphotropic virus associated with a mild viral disease encountered during childhood. Nonetheless, it has been observed in adulthood, especially among immunocompromised individuals [5]. HHV-6 infection can present with a wide range of symptoms, from isolated fevers to myalgias, exanthems, and even neurological symptoms including seizures or headaches [10]. According to the literature, HHV-6 has been implicated in the development of multiple neurological disorders including multiple sclerosis, encephalitis, and mesial temporal lobe epilepsy; it is believed that the nasal cavity is a pathway of entry to the central nervous system by the virus [11]. 

Despite being unusual, there have been some reported cases of HHV-6 meningitis in immunocompetent adults [12]. Some studies have established a link between HHV-6 and Hashimoto thyroiditis, an autoimmune endocrine illness; however, no direct link has been found to other autoimmune illnesses [13]. Despite this association with thyroiditis, there are no studies establishing a direct correlation between having a history of psoriasis and the development of CNS infections by HHV-6 in adults. Some studies were able to isolate cytomegalovirus and human herpes virus 7 in the skin of patients with chronic plaque psoriasis, suggesting a possible correlation of some herpesviruses with the occurrence of psoriatic plaques [14]. Although no study was able to suggest a direct connection between the presence of psoriasis and the increased incidence of viral illnesses, given the absence of comparable findings in other cases, it is possible that the presence of psoriasis might have been incidental.

The above report describes the case of a patient with a past medical history significant for plaque psoriasis presenting to the hospital with persistent headaches. Thorough workup yielded positive PCR for HHV-6 in the CSF fluid leading to a diagnosis of meningitis secondary to this organism. The suspected exposure for HHV-6 is likely from exposure to sick contact in the household. While reactivation of HHV-6 is well established among the severely immunocompromised population, this is not the case for immunocompetent individuals with skin-related rheumatoid diseases [15]. Studies have shown that there is a correlation between plaque psoriasis and herpesviruses, which suggests a possible link between the two conditions [14]. This finding raises the question of whether there is a connection between the resurfacing of the virus or the plaques themselves and plaque psoriasis. Nevertheless, with advancements in laboratory techniques and the increasing use of PCR, there is a rise in the detection of various organisms that would have otherwise not been isolated.

## Conclusions

The importance of this case resides in highlighting the occurrence of HHV-6 infections in people with underlying rheumatologic skin disorders. It is crucial to further investigate the link between psoriasis and the emergence of various herpesviruses in immunocompetent adults.
